# Towards a more cognitive evaluation of progressive aphasias?

**DOI:** 10.1093/braincomms/fcac268

**Published:** 2022-10-21

**Authors:** Antoine Renard, Jean-Marie Annoni

**Affiliations:** Unité de recherche PsyNCog, Faculté de Psychologie, Logopédie et Sciences de l’éducation, Université de Liège, 4000 Liège, Belgium; Laboratory of Cognitive and Neurological Sciences, Department of Neuro and Mouvement Sciences, Faculty of Science and Medicine, University of Fribourg, 1700 Fribourg, Switzerland

## Abstract

This scientific commentary refers to ‘Unclassified fluent variants of primary progressive aphasia: distinction from semantic and logopenic variants’ by Watanabe *et al*. (https://doi.org/10.1093/braincomms/fcac015)

**This scientific commentary refers to ‘Unclassified fluent variants of primary progressive aphasia: distinction from semantic and logopenic variants’ by Watanabe et al. (https://doi.org/10.1093/braincomms/fcac015)**

With their well-described multiple case study in *Brain Communications*, Watanabe *et al*. have brought a timeliness argument for heterogeneity of neurodegenerative language impairment.^1^ During a 3-year period in two large tertiary hospitals, they prospectively included and clinically and neuropsychologically described 12 patients, who satisfied the diagnostic criteria of fluent primary progressive aphasia (PPA) but did not fulfil the classical criteria of either semantic or logopenic variant.^2^ Based on classical taxonomy that is also used in ‘vascular’ aphasias, they found clinical features of anomic aphasia (*n* = 8), primary progressive transcortical sensory aphasia (*n* = 3) and primary progressive Wernicke’s aphasia (*n* = 1). One strength of the study is the apparent early phase of the disease (CDR 0.5, disease evolution between 2 and 5 years) and similar schooling (12–16 years) of the patients. Although not explicitly mentioned, there appeared to be no signs of vascular lesions in the patients’ MRIs. The authors discussed the aphasia features and the brain atrophy pattern of these non-canonical fluent types. Concerning the pattern of atrophy, anomic patients tend to have bilateral temporoparietal atrophy, while the temporoparietal atrophy was lateralized to the left in the other groups (with comprehension impairment). There is no biomarker analysis, so more precise neuropathological dementia aetiology is not possible. However, MRI revealed significant entorhinal or hippocampal atrophy raising the question of whether such progressive aphasia is ‘established’ PPA, part of the frontotemporal dementias, or other pathologies, such as corticobasal degeneration, synucleinopathies or atypical Alzheimer’s disease. The authors also discuss this issue, arguing the importance of clinical classification.

The diagnosis of PPA is a scientific challenge due to clinical, radiologic and pathophysiological heterogeneity,^3^ 11 years after the consensus criteria proposed by Gorno-Tempini *et al*.^2^ This seminal paper has allowed the inclusion criteria for the three variants to be defined: semantic, logopenic and non-fluent. However, it has always been accepted that these variants might be clinically and scientifically relevant but not necessarily biologically exhaustive.

For the last few decades, research has made it possible to refine variants and propose new ones. For example, Gorno-Tempini *et al*. proposed the logopenic variant as a new entity that is characterized by a central phonological impairment. In the same period, Adlam *et al*.^4^ considered fluent progressive aphasia and semantic dementia as ‘two sides of the same coin’. Meantime, Rohrer *et al*.^5^ proposed a conceptual framework for the analysis of word-finding difficulties in clinical practice for PPA patients.

The first focus of the Watanabe *et al*. paper is a detailed description of linguistic impairment and the current criteria of PPA. The language assessment they conducted did not allow them to classify 12 patients among the 3 consensual variants. In particular, they could demonstrate a high number of anomic cases with little semantic or phonological impairment and with a bilateral pattern of brain atrophy.

The concept of borderline clinical features is not new. In clinical practice, it is common to not be able to classify patients in the consensual variants proposed. Classification difficulties and the limits of the criteria of Gorno-Tempini^2^ were *de facto* quickly raised^6^ after 2011. Patients who do not meet the nf/a or sv criteria are not necessarily *de facto* a logopenic variant and remain unclassifiable. In a recent study of syntactic deviations in PPA, one-sixth of the patients fall outside the three classical variants.^7^ The primary interest in the study by Watanabe is specific focus on these borderline clinical pictures.

Another interesting aspect of the paper is that the authors propose strategies to overcome these classification-induced issues. Quantitative classification in the early and mild stages of PPA, especially semantic variants, is challenging. There is evidence for heterogeneity of the linguistic profiles of PPA on the one hand and their pathophysiological correspondences on the other hand.^3^ This case study provides arguments to improve current clinical criteria. The description of the present paper aims to name these unusual cases of primary fluent aphasia, proposing the use of the terms anomic, transcortical sensory and Wernicke aphasia.

This article has raised several questions for us regarding its limitations, and we would like to argue beyond the current limitations of consensual criteria and a certain number of theoretical and methodological limitations. We would like to raise certain issues, which could be interesting to consider in future research.

The first issue is the use of semiological tests from vascular aphasiology. Cadório *et al*. demonstrated the absence of current theoretical foundations, psycholinguistic variables as well as psychometric and normative weaknesses of the tests, particularly when dealing with neurodegenerative aphasia.^8^ Their diagnostic accuracy and the cognitive interpretation of language disturbances are generally limited. Second, a clinical analysis of the disorders from the aphasic semiological framework does not necessarily bring an improvement in itself. Indeed, the semiological classifications of aphasias could be reinterpreted, and has a low classification power,^9^ which is no higher than the current criteria of PPA. Third, Ingram *et al*.^10^ prospectively included 46 PPA patients; from a comprehensive assessment, they suggest that a multidimensional (semantic, phonology, executive) and continuous rather than categorical and discrete classification system may be a better conceptualization of aphasia, whatever the aetiology.

Finally, these patients, although apparently at the early and mild stages, may already be in a more advanced phase, particularly in fluent variants.^11^ This could have significant consequences on language profiles, especially for the semantic variant.^12^ Indeed, the comprehension disorder is a cardinal point in the diagnosis of this variant. Moreover, the use of an insufficiently sensitive test can give a ‘false negative’ type result and lead to the classification of patients as logopenic or fluent progressive aphasia.

Considering all of these elements, and integrating the careful analysis of the authors, the present analysis provides arguments for a more open clinical classification. Moreover, future research should include more patients and advanced language assessments based on theoretically motivated tests, including non-verbal semantics as well as more items.

The performance analysis should be done in a multidimensional way and from continuous variables and no longer dichotomized.^10^

Finally, the cognitive approach could constitute a powerful, reliable and novel biomarker to improve the diagnosis and classification of PPA.^13^ Thus, by going beyond the symptoms and determining the neurocognitive origin of linguistic disturbances, it would allow a robust and meaningful classification, like the pathophysiological one. The use of cognitive classification is certainly the missing piece of a puzzle that is just waiting to be completed.

To conclude, this article raises the delicate question of classification and of the patients who find themselves at the limits of these, not fully responding to either one or the other variant. Therefore, either the scientific community accepts our neurobiological complexity by tolerating ‘borderline’ situations or experts will have to add as many variants as necessary, at the risk, however, of ending up with a large number of PPA types, but ultimately rarely observed in clinical practice. Watanabe *et al*.^1^ in this matter leave the different possibilities open at this time.

## Data Availability

Data sharing is not applicable to this article as no new data were created or analysed.
